# Artificial-Intelligence-Based Radiologic, Histopathologic, and Molecular Models for the Diagnosis and Classification of Malignant Salivary Gland Tumors: A Systematic Review and Functional Meta-Synthesis

**DOI:** 10.3390/medsci14020183

**Published:** 2026-04-05

**Authors:** Carlos M. Ardila, Eliana Pineda-Vélez, Anny M. Vivares-Builes, Alejandro I. Díaz-Laclaustra

**Affiliations:** 1 Department of Periodontics, Saveetha Dental College and Hospitals, Saveetha Institute of Medical and Technical Sciences, Saveetha University, Chennai 600077, India; 2 Biomedical Stomatology Research Group, Basic Sciences Department, Faculty of Dentistry, Universidad de Antioquia (U. de A.), Medellín 050010, Colombia; eliana.pineda@uam.edu.co (E.P.-V.); anny.vivares@uam.edu.co (A.M.V.-B.); 3 Faculty of Dentistry, Institución Universitaria Visión de las Américas, Medellín 050040, Colombia; 4 Department of Basic Sciences, Faculty of Dentistry, Universidad de Antioquia, Medellín 050040, Colombia; isbet.diaz@udea.edu.co

**Keywords:** salivary gland neoplasms, artificial intelligence, machine learning, radiomics, DNA methylation

## Abstract

Background/Objectives: Malignant salivary gland tumors (MSGTs) are rare, biologically heterogeneous neoplasms in which histopathologic diagnosis and classification are challenging and subject to interobserver variability. Artificial intelligence (AI) approaches using radiologic, histopathologic, and molecular data, including radiomics, deep learning, and biomarker-based models, have been proposed as adjunctive diagnostic tools. This systematic review aimed to identify and critically appraise AI/ML models across radiologic, histopathologic, and molecular domains for distinct diagnostic tasks in MSGTs, and to integrate their diagnostic roles through a functional meta-synthesis. Methods: We conducted a PRISMA 2020-compliant systematic review. Embase, PubMed/MEDLINE, and Scopus were searched from inception to February 2026. Eligible studies developed or validated AI/ML diagnostic or classification models in human salivary gland tumor cohorts and reported extractable performance metrics. Results: From 1265 records, eight studies (1922 participants) met the inclusion criteria, spanning CT/MRI radiomics or deep learning (n = 4), whole-slide histopathology deep learning (n = 3), and DNA methylation-based classification (n = 1). External validation was reported in two CT-based benign–malignant discrimination studies, with AUCs of 0.890 (95% CI 0.844–0.937) and 0.745 (95% CI 0.699–0.791). Heterogeneity in model construction, outcome definitions, and validation strategies precluded meta-analysis. Risk of bias was frequently high in QUADAS-2/PROBAST assessments, driven by retrospective sampling, limited blinding, and analysis-related concerns, while calibration and utility were rarely assessed. Conclusions: AI/ML models for MSGTs demonstrate promising diagnostic performance, particularly for preoperative benign–malignant discrimination, but the current evidence base is limited by heterogeneity, predominantly internal validation, and high risk of bias. The functional meta-synthesis identified three convergent diagnostic domains: malignancy discrimination, histopathologic subtype classification, and molecular/epigenetic taxonomy refinement.

## 1. Introduction

Malignant salivary gland tumors (MSGTs) are rare but biologically diverse neoplasms that account for less than 5% of head and neck malignancies. Despite their low incidence, they comprise more than twenty malignant epithelial entities according to the current World Health Organization classification, each characterized by distinct morphological, immunophenotypic, and molecular features [1]. Their heterogeneity, overlapping histologic patterns, and variable clinical behavior make accurate diagnosis challenging, with important implications for treatment selection and prognosis.

Histopathologic assessment remains the diagnostic cornerstone; however, substantial interobserver variability has been reported, particularly in tumors with basaloid or myoepithelial differentiation and in low-grade carcinomas with subtle invasive features [2,3]. Even well-established entities may not be fully captured by conventional morphology alone, underscoring the need for more objective adjunctive diagnostic tools.

Molecular pathology has improved classification through the identification of recurrent gene fusions such as ETV6-NTRK3 in secretory carcinoma and MYB/MYBL1 rearrangements in adenoid cystic carcinoma [4,5]. However, these alterations are not universally present, and several tumor types lack specific molecular hallmarks, leaving a proportion of cases diagnostically uncertain.

Epigenetic profiling, particularly DNA methylation analysis, has emerged as a robust classification approach. These signatures are highly tissue-specific and stable, enabling machine-learning-based algorithms to classify tumors according to global epigenetic patterns [6,7]. Jurmeister et al. [2] demonstrated that methylation-based classification can achieve high diagnostic accuracy and identify biologically relevant subgroups.

Parallel developments in digital pathology and radiomics have further expanded the diagnostic landscape. Convolutional neural networks have shown promising performance in histopathologic tumor classification [3,8,9], while radiomics enables the extraction of high-dimensional imaging features beyond visual interpretation [10]. MRI-based radiomic models have demonstrated encouraging performance in differentiating benign and malignant parotid tumors [11,12,13]. Nevertheless, variability in datasets, feature extraction, and validation strategies limits generalizability across studies.

Despite these advances, the available evidence remains fragmented across imaging, histopathologic, and molecular domains. Most studies are modality-specific, based on small retrospective cohorts, and rely on internal validation, with inconsistent reporting standards.

Importantly, no prior work has systematically integrated these heterogeneous artificial intelligence (AI) approaches within a unified interpretative framework. The existing literature is largely performance-driven and lacks a higher-order synthesis that clarifies how different AI modalities contribute to diagnostic reasoning.

In this context, functional meta-synthesis refers to an interpretative integration of heterogeneous diagnostic AI models into abstracted functional domains rather than a purely statistical aggregation of performance outcomes.

The present study, therefore, aims to systematically identify and critically appraise AI and machine-learning models applied to radiologic imaging, digital histopathology, and molecular biomarkers for distinct diagnostic tasks in malignant salivary gland tumors. Additionally, we aim to integrate their roles through a functional meta-synthesis. By synthesizing these approaches within a unified conceptual framework, this study seeks to clarify their complementary diagnostic functions and to define future directions for clinical translation.

## 2. Materials and Methods

This systematic review and functional meta-synthesis were conducted in accordance with the Preferred Reporting Items for Systematic Reviews and Meta-Analyses (PRISMA 2020) statement [14]. The protocol was developed a priori and registered in the International Prospective Register of Systematic Reviews (PROSPERO; CRD420261327571).

### 2.1. Eligibility Criteria

Eligibility criteria were defined according to a PICOS framework. The review question was formulated as follows: In patients with malignant salivary gland tumors or in cohorts designed to differentiate benign from malignant salivary gland neoplasms (P), how do AI-based radiomic, digital histopathologic, or molecular biomarker models (I), compared with conventional diagnostic approaches or alternative modeling strategies (C), perform in terms of diagnostic or classification accuracy (O) in original model development or validation studies (S)?

#### 2.1.1. Inclusion Criteria

Studies were eligible if they involved human participants with malignant salivary gland tumors or cohorts specifically designed to differentiate benign from malignant salivary gland neoplasms. Eligible studies evaluated AI or machine-learning models applied to radiomic imaging features (e.g., magnetic resonance imaging [MRI] and computed tomography [CT]), digital histopathology (including whole-slide imaging), or molecular and biological biomarkers, such as DNA methylation profiles or genomic markers. Comparator approaches could include conventional clinical, radiologic, or histopathologic assessment, traditional statistical models, or alternative AI strategies. Only diagnostic or classification model development or validation studies reporting performance metrics—such as area under the curve (AUC), sensitivity, specificity, accuracy, balanced accuracy, positive predictive value, or negative predictive value—were included. Studies conducted in any clinical or geographic setting were considered eligible.

#### 2.1.2. Exclusion Criteria

Studies focusing exclusively on benign tumors without a malignancy-related endpoint were excluded. Animal experiments, in vitro studies, and investigations involving mixed head and neck tumor cohorts without extractable salivary gland-specific data were also excluded. Studies limited to segmentation tasks without predictive modeling, non-algorithmic imaging analyses, or purely descriptive approaches were excluded. Reviews, editorials, conference abstracts without extractable data, and case reports lacking model evaluation were likewise excluded.

### 2.2. Information Sources and Search Strategy

A comprehensive search strategy was implemented across Embase (via Ovid), PubMed/MEDLINE, and Scopus from database inception to February 2026 without language or date restrictions. Only published studies were sought. Search terms combined controlled vocabulary, including MeSH terms such as “Salivary Gland Neoplasms,” “Artificial Intelligence,” “Machine Learning,” “Radiomics,” and “Biomarkers,” with free-text keywords related to malignant salivary gland tumors, radiomic analysis, digital pathology, DNA methylation, and diagnostic classification models. Additional records were identified through manual reference list screening, forward citation tracking, and expert consultation when necessary. The complete search strategies for all databases are provided in Supplementary Table S1.

### 2.3. Study Selection

Titles and abstracts were independently screened by two reviewers with experience in oral pathology and evidence-based research, followed by full-text assessment of potentially eligible studies. Inter-reviewer agreement was high (κ = 0.91). Disagreements were resolved through discussion and consensus, and when required, a third reviewer adjudicated. Consensus was required in a minority of cases, primarily involving studies with unclear eligibility criteria. The selection process was documented using a PRISMA flow diagram.

### 2.4. Data Extraction

Data extraction was performed independently by two reviewers with experience in oral pathology and evidence-based research, using a standardized, prepiloted data collection form. Inter-reviewer agreement was high (κ = 0.91), indicating strong consistency in the extraction process. When necessary, the study authors were contacted for clarification or to address missing information. Extracted variables included study identification details, study design and setting, sample size and population characteristics, tumor entities included, type of AI or machine-learning algorithm, input modality such as radiomics, digital histology, or molecular biomarkers, feature extraction methodology, validation strategy including internal and external validation, performance metrics, calibration and clinical utility measures, interpretability techniques, and reporting adherence. Disagreements were infrequent and resolved through discussion and consensus, with a third reviewer involved when needed. Consensus was required in a minority of cases, primarily in studies with incomplete reporting or complex model descriptions.

### 2.5. Risk of Bias and Reporting Quality Assessment

Methodological quality and risk of bias were evaluated using instruments appropriate to diagnostic and predictive modeling research. For diagnostic accuracy studies, the QUADAS-2 tool was applied [15], and where applicable, the QUADAS-AI extension was considered [16]. For prediction or classification modeling studies beyond classical diagnostic accuracy frameworks, the Prediction Model Risk of Bias Assessment Tool (PROBAST) was used [17]. Reporting transparency was evaluated in accordance with TRIPOD guidance and emerging TRIPOD-AI recommendations [18]. Risk of bias assessments were conducted independently by two reviewers and summarized narratively and in tabular format.

### 2.6. Assessment of Reporting Bias

When at least ten clinically comparable studies addressed the same diagnostic question with external validation data, publication bias was planned to be assessed using Deeks’ funnel plot asymmetry test [19]. When quantitative pooling was not feasible, qualitative analysis of reporting bias was conducted.

### 2.7. Certainty of Evidence

Certainty of evidence was evaluated using the GRADE framework adapted for diagnostic test accuracy studies [20]. The domains assessed included risk of bias, inconsistency, indirectness, imprecision, and publication bias. Evidence certainty was categorized as high, moderate, low, or very low. When quantitative synthesis was not possible, a structured narrative certainty assessment was performed.

### 2.8. Data Synthesis and Functional Meta-Synthesis

In addition to descriptive synthesis, a functional meta-synthesis was conducted using an inductive interpretative framework. To improve transparency and reproducibility, the process was structured in sequential stages.

First, two reviewers independently characterized each included study according to three predefined dimensions: (i) primary diagnostic objective, (ii) input modality (imaging, histopathologic, or molecular), and (iii) level of biological abstraction.

Second, an initial coding framework was developed iteratively based on recurring diagnostic objectives identified during data extraction. Studies were then independently coded within these evolving functional categories, with flexibility to refine categories as new patterns emerged.

Third, studies were grouped by shared functional roles rather than by performance metrics. Domain assignment followed predefined decision rules, whereby each study was classified according to its primary clinical function, defined by the intended diagnostic task and the level of biological inference (macroscopic imaging, microscopic histopathology, or molecular classification). When multiple objectives were present, classification was based on the dominant endpoint as defined by the study authors. Discrepancies were resolved through consensus discussion.

Fourth, a constant comparative approach was applied to enhance internal consistency. Newly identified diagnostic functions were iteratively contrasted with previously coded studies until stable domain categories were achieved.

Finally, the functional domains were derived through cross-modal comparison and abstraction of recurrent diagnostic patterns across heterogeneous AI systems. This step enabled interpretative integration beyond conventional narrative synthesis by explicitly linking model function to clinical application.

Quantitative meta-analysis was planned only when at least two studies provided external test-set data addressing the same diagnostic question with sufficiently comparable outcome definitions. In the absence of sufficient homogeneity, findings were synthesized narratively with emphasis on external validation performance, heterogeneity, and translational applicability.

## 3. Results

### 3.1. Study Selection Process

**Figure 1:**
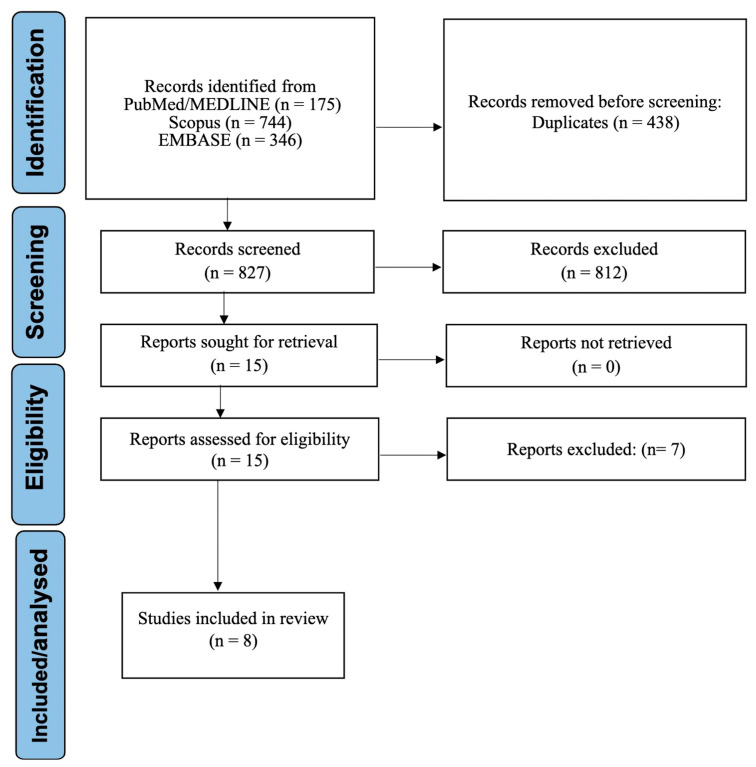
PRISMA 2020 flow diagram illustrating the study selection process.

The study selection process is summarized in the PRISMA flow diagram (Figure 1). A total of 1265 records were initially identified across the searched databases. After removing duplicates and screening titles and abstracts using the prespecified eligibility criteria, 15 full-text articles were assessed for eligibility. Following full-text evaluation, eight studies met all inclusion criteria and were included in the systematic review and functional meta-synthesis [2,3,21,22,23,24,25,26]. Seven studies were excluded at the full-text stage due to (i) lack of extractable salivary gland-specific data within mixed head and neck tumor cohorts, (ii) absence of diagnostic or classification performance metrics despite reporting AI-based image analysis, and (iii) focus on segmentation or feature extraction tasks without implementation of a predictive or classification model.

### 3.2. Descriptive Characteristics of Included Studies

The eight included studies comprised a total of 1922 human participants, spanning multiple diagnostic modalities and AI/ML pipelines (Table 1). Four studies evaluated imaging-based radiomic/deep-learning models for differentiating benign versus malignant parotid tumors or for multi-class parotid tumor classification using CT or MRI [21,22,23,24]. Two studies assessed whole-slide image (WSI) deep-learning models for histopathologic discrimination of malignant salivary gland entities or malignancy-related differentials [25,26], and one additional study evaluated CNN-based classification on digitized histopathology slides from salivary gland carcinoma specimens [3]. One study investigated DNA methylation-based machine-learning classification across a multi-entity salivary gland tumor cohort [2].

The included studies addressed distinct and non-equivalent diagnostic tasks, including benign–malignant discrimination, histopathologic tumor subtype classification, and molecular or epigenetic taxonomy refinement. These tasks differ in clinical purpose and level of biological inference and were therefore not directly comparable; they were instead analyzed within a unified functional framework.

Across imaging-based cohorts, patient-level labels were histopathologically confirmed, and the dominant design was retrospective with internal splitting into training and test subsets; multicenter external testing was available in two CT-based studies [22,23]. Across histopathology-based deep-learning studies, WSIs were used for patch-level training and test evaluation with performance reported on held-out subsets rather than independent external cohorts [25,26]. The methylation-based study trained and evaluated a multi-class classifier using repeated cross-validation and extended the conventional classification by identifying epigenetically distinct entities/subgroups [2].

### 3.3. External Validation Performance (Primary Outcome)

Two studies reported performance in an external testing cohort for the benign–malignant discrimination task using CT-based AI models [22,23]. Yu et al. [22] evaluated six deep-learning architectures on contrast-enhanced CT arterial-phase images and reported MobileNet V3 as the best-performing model. In the external-testing set, MobileNet V3 achieved an AUC of 0.890 (95% CI 0.844–0.937) with accuracy 0.846, sensitivity 0.828, and specificity 0.860 (PPV 0.716; NPV 0.917). Shen et al. [23] developed CT radiomic models incorporating intratumoral and peritumoral features and selected the Tumor + External2 radiomics signature, implemented with SVM, as the best radiomic model. In the external-testing cohort, the radiomics model achieved an AUC of 0.745 (95% CI 0.699–0.791), with accuracy of 0.773, sensitivity of 0.794, and specificity of 0.714. The combined clinical–radiomic model in the same external cohort showed an AUC of 0.749 (95% CI 0.705–0.793), with the same accuracy and sensitivity reported for that cohort (accuracy 0.773; sensitivity 0.794; specificity 0.714). Overall, externally tested CT-based AI models showed moderate-to-high discrimination for benign–malignant parotid tumor differentiation, although performance varied across cohorts and model constructions [22,23].

Quantitative pooling was not conducted because only two studies provided external validation for a comparable benign–malignant discrimination task, and substantial heterogeneity was observed in index test construction, feature extraction pipelines, outcome definitions, and validation frameworks, precluding statistically meaningful aggregation.

A structured summary of externally and internally validated diagnostic performance metrics across studies is presented in Table 2, with 95% confidence intervals reported when available and explicitly noted as absent when absent in the original studies.

### 3.4. Internal Validation or Cross-Validation Performance (Secondary Outcomes)

In MRI-based radiomics, He et al. [21] developed a three-step machine-learning framework (XGBoost/SVM/DT) to classify parotid neoplasms into four subtypes. The study reported stepwise AUCs and confusion-matrix accuracies for the four-class task. XGBoost yielded the highest AUCs in the training cohort across the three steps (0.857, 0.882, 0.908), and in the test cohort for step 1 (AUC 0.826). In Steps 2 and 3 of the test cohort, SVM slightly outperformed XGBoost (AUC 0.833 vs. 0.817; and 0.821 vs. 0.789, respectively). For the final four-class confusion-matrix task in the test cohort, XGBoost and SVM achieved total accuracies of 70.8% and 59.6%, respectively, exceeding the radiologist’s accuracy reported for the same task (49.2%). Committeri et al. [24] evaluated MRI radiomics and inflammatory biomarkers in 117 patients (47 with Warthin tumors, 42 with pleomorphic adenomas, and 28 with malignant carcinomas). After univariate feature selection, the best-performing multivariate model was an SVM using six features (two radiomics metrics plus four clinical inflammatory indices), achieving 86% accuracy, 68% sensitivity, and 91% specificity on the test dataset, with ROC and confusion-matrix outputs presented by the authors.

For histopathology-based AI, Schulz et al. [3] digitized 118 histological slides from 68 patients and compared four CNN architectures across multiple pixel sizes. The authors reported accuracy values ranging from 18.8% to 84.7%, with Inception v3 achieving the highest accuracy at 500 × 500 pixels; recall/sensitivity reached up to 85% across architectures and pixel sizes. Sousa-Neto et al. [25] investigated deep learning on WSIs for challenging salivary gland differentials. In carcinoma ex pleomorphic adenoma (CXPA) versus pleomorphic adenoma (PA), using WSIs from 83 patients, the ResNet-50 test performance reported accuracy (total) 0.93, sensitivity 0.94, specificity 0.88, F1 score 0.95, and AUC 0.97. In acinic cell carcinoma (AciCC) versus secretory carcinoma (SC), using 54 WSIs from 46 patients, InceptionV3 showed the best overall test performance (accuracy total 0.81; sensitivity 0.90; specificity 0.73; F1 score 0.81), while the highest AUC reported among the evaluated networks was 0.86 (VGG16) [26].

Finally, Jurmeister et al. [2] analyzed DNA methylation profiles from a cohort of 363 cases covering 20 salivary gland tumor entities and developed a calibrated SVM-based classifier evaluated via repeated cross-validation. The authors reported a mean balanced accuracy of 0.991 and highlighted that methylation profiling supported and expanded conventional tumor classification by identifying epigenetically distinct entities and relevant subgroups.

### 3.5. Functional Meta-Synthesis

**Figure 2:**
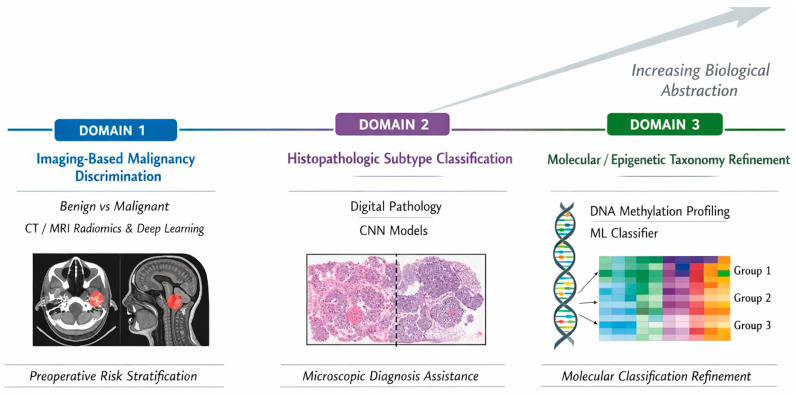
Functional Domains of Artificial Intelligence Applications in Malignant Salivary Gland Tumors. Conceptual representation of the three functional domains identified in the meta-synthesis. Domain 1 (Imaging-Based Malignancy Discrimination) includes CT- and MRI-based radiomic or deep-learning models differentiating benign from malignant tumors [21,22,23,24]. Domain 2 (Histopathologic Subtype Classification) encompasses convolutional neural network models applied to whole-slide images for subtype discrimination [3,25,26]. Domain 3 (Molecular/Epigenetic Taxonomy Refinement) represents DNA methylation-based machine-learning classification systems refining tumor taxonomy [2]. The domains illustrate increasing biological abstraction from macroscopic imaging assessment to molecular stratification.

The functional meta-synthesis integrated heterogeneous AI models into conceptual diagnostic domains based on their primary clinical function rather than solely on their performance metrics. This approach does not assume equivalence between tasks, but rather seeks to integrate them as complementary components of diagnostic reasoning across different biological and clinical levels. Across the eight included studies, three overarching functional domains emerged: malignancy discrimination, tumor subtype classification, and molecular or epigenetic taxonomy refinement. These domains are conceptually summarized in Figure 2.

#### 3.5.1. Domain 1—Malignancy Discrimination (Benign vs. Malignant)

Four studies primarily addressed binary discrimination between benign and malignant salivary gland tumors using imaging-based or hybrid models [21,22,23,24]. CT-based deep-learning models demonstrated externally validated discrimination in multicenter settings. Yu et al. [22] reported an external AUC of 0.890 with balanced sensitivity (0.828) and specificity (0.860), indicating stable discrimination across institutions. Shen et al. [23] reported an externally validated AUC of 0.745 for radiomics-based SVM classification, reflecting more modest performance compared with end-to-end convolutional architectures. MRI-based radiomics [21] and hybrid radiomics–biomarker models [24] demonstrated promising diagnostic discrimination in internally validated cohorts but lacked independent external validation. In several of these models, specificity exceeded sensitivity, suggesting a conservative tendency in malignant classification.

Functionally, models within this domain operate as preoperative risk-stratification systems, supporting radiologic decision-making and potentially influencing surgical planning and biopsy strategy.

#### 3.5.2. Domain 2—Histopathologic Tumor Subtype Classification

Three studies evaluated deep-learning approaches applied to digitized whole-slide histopathology images [3,25,26]. Schulz et al. [3] compared convolutional neural network architectures for salivary gland carcinoma classification and achieved accuracy values up to 84.7%, depending on resolution and model design. Sousa-Neto et al. [25] reported an AUC of 0.97 in differentiating carcinoma ex pleomorphic adenoma from pleomorphic adenoma, while Sousa-Neto et al. [26] reported AUC values up to 0.86 for the differentiation of acinic cell carcinoma and secretory carcinoma.

These models are intended as microscopic decision-support approaches; however, their clinical applicability remains uncertain due to the lack of external validation and real-world testing.

#### 3.5.3. Domain 3—Molecular and Epigenetic Taxonomy Refinement

Jurmeister et al. [2] applied genome-wide DNA methylation profiling combined with machine learning to classify 363 salivary gland tumors across 20 entities. The classifier achieved a mean balanced accuracy of 0.991 under cross-validation. Beyond diagnostic categorization, methylation profiling revealed biologically meaningful subgroups. It clarified disputed entities, suggesting a potential to refine tumor taxonomy at a molecular level. However, evidence is currently limited to internally validated settings.

This domain differs fundamentally from imaging and histopathology applications, as it contributes not only to diagnostic support but also to biologically grounded reclassification frameworks.

#### 3.5.4. Cross-Domain Observations

When examined collectively, the studies reveal a hierarchical progression of AI applications across diagnostic depth. Imaging-based models predominantly operate at the macroscopic level and assist in preoperative malignancy risk estimation. Histopathologic deep-learning systems function at the microscopic level, supporting subtype discrimination within established morphologic frameworks. In contrast, methylation-based machine-learning models operate at a molecular systems level and contribute to the redefinition and refinement of tumor classification.

External validation was limited to selected CT-based imaging studies [22,23], whereas histopathologic and epigenetic classifiers relied primarily on internal validation or cross-validation strategies. Considerable heterogeneity was observed in feature extraction pipelines, model architectures, outcome definitions, and reporting of performance metrics, which precluded quantitative pooling across modalities.

Taken together, the functional meta-synthesis demonstrates that AI in malignant salivary gland tumors does not represent a single diagnostic tool but rather a spectrum of systems operating across complementary biological and clinical layers. Accordingly, performance metrics should be interpreted within the context of each specific diagnostic task rather than compared across domains.

### 3.6. Additional Model Characteristics: Predictive Values, Calibration, Clinical Utility, and Interpretability

#### 3.6.1. Positive and Negative Predictive Values

The reporting of predictive values was heterogeneous across studies. Explicit positive predictive value (PPV) and negative predictive value (NPV) were reported in the externally validated CT-based deep-learning study by Yu et al. [22], in which the model achieved a PPV of 0.716 and an NPV of 0.917 in an external testing cohort. Similarly, Shen et al. [23] reported PPV and NPV for the external-testing cohort; for the selected best radiomic signature (Tumor + External2), PPV was 0.885, and NPV was 0.555. Committeri et al. [24] reported PPV and NPV primarily in the context of individual clinical or radiologic parameters; for example, the neutrophil-to-lymphocyte ratio demonstrated a PPV of 1.0 and an NPV of 0.86. However, predictive values were not consistently reported for the final multivariable SVM model. Sousa-Neto et al. [25,26] reported negative predictive value in their deep-learning analyses, but did not consistently report PPV; instead, precision was provided as a performance metric. In contrast, He et al. [21], Schulz et al. [3], and Jurmeister et al. [2] did not explicitly report PPV or NPV.

Overall, predictive values were available for a subset of imaging-based and histopathologic studies but were not uniformly reported across modalities, limiting the comparative interpretation of post-test probabilities.

#### 3.6.2. Calibration Metrics

Formal calibration assessment was rarely performed. Shen et al. [23] explicitly reported calibration curves and Brier scores, demonstrating satisfactory agreement between predicted and observed probabilities. This represents the only study among the included cohort to provide explicit quantitative calibration metrics. Jurmeister et al. [2] described a probabilistic calibration procedure within the methylation classifier development pipeline, but did not report calibration curves, Brier scores, or other calibration performance measures as outcome results. The remaining studies, including Yu et al. [22], He et al. [21], Committeri et al. [24], Schulz et al. [3], and Sousa-Neto et al. [25,26], did not report formal calibration metrics.

Thus, while discrimination was frequently emphasized, probability calibration—essential for clinical implementation—was largely underreported.

#### 3.6.3. Clinical Utility and Decision Curve Analysis

Assessment of clinical utility was limited to a single study. Shen et al. [23] performed decision curve analysis and reported net benefit across a range of threshold probabilities, demonstrating improved clinical utility of the combined clinical–radiomics model compared with radiomics alone. No other included study reported decision curve analysis or net benefit evaluation. Although Yu et al. [22] provided external validation metrics, no formal clinical utility analysis was presented. Similarly, the MRI-based radiomics study by He et al. [21], the biomarker-integrated model by Committeri et al. [24], the deep-learning histopathology models [3,25,26], and the methylation classifier [2] did not include decision curve analysis.

Consequently, despite promising discrimination performance, formal quantification of potential impact on clinical decision-making remains limited.

#### 3.6.4. Interpretability Methods

Model interpretability approaches varied across modalities. Yu et al. [22] explicitly implemented Gradient-weighted Class Activation Mapping (Grad-CAM) to visualize image regions contributing to classification decisions, providing a transparent explainability framework for the deep-learning model. Radiomics-based studies, including He et al. [21] and Shen et al. [23], described feature extraction and selection processes, enabling indirect interpretability through ranked radiomic features, although no SHAP-based or gradient-based explainability tools were reported. Committeri et al. [24] similarly identified relevant clinical and radiologic parameters within the modeling process but did not implement formal explainability algorithms.

Jurmeister et al. [2] provided interpretability at a biological systems level, correlating methylation clusters with established histopathologic entities and identifying biologically meaningful subgroups. Histopathologic deep-learning studies [3,25,26] focused primarily on performance metrics and did not report formal activation mapping or explainability tools within the published analyses.

Collectively, explicit explainability methods were limited, with Grad-CAM implementation reported only in Yu et al. [22], while other studies relied on feature transparency or biological interpretability rather than on algorithmic explainability frameworks.

### 3.7. Risk of Bias Assessment

Risk of bias was evaluated using QUADAS-2 [15] for diagnostic accuracy domains and PROBAST [17] for prediction model domains. Detailed domain-level judgments are summarized in Table 3 and Table 4.

Across studies, patient selection was frequently judged to be high risk due to the retrospective design and non-consecutive sampling. However, important study-level differences were noted. For example, in Jurmeister et al. [2], the use of a multicenter cohort with heterogeneous tumor entities may introduce concerns regarding case-mix representativeness and potential selection bias. In contrast, imaging-based studies such as Yu et al. [22] and Shen et al. [23], although also retrospective, partially mitigated applicability concerns through multicenter external validation cohorts.

Index test bias was commonly rated as high, primarily due to insufficient reporting of blinding procedures and lack of prespecified thresholds. In histopathologic deep-learning studies [3,25,26], limited reporting of data partitioning procedures and model development steps, as well as reduced transparency in model tuning, raise potential concerns regarding overfitting and optimistic performance estimates.

The reference standard domain was generally considered low risk because histopathologic confirmation served as the diagnostic ground truth. However, in molecular classification studies such as Jurmeister et al. [2], the use of methylation-based clustering as both a classification tool and a reference framework may introduce conceptual overlap, potentially affecting independence between the index test and the reference standard.

Flow and timing were often rated as unclear due to incomplete reporting of exclusions and missing data handling. Under PROBAST, recurrent concerns were identified in the participants and analysis domains. Limited reporting of feature selection procedures, the absence of external validation, and the lack of calibration assessment raise concerns about generalizability and model reliability.

### 3.8. Reporting Transparency (TRIPOD/TRIPOD-AI)

Overall reporting transparency across the eight included studies was variable, with important differences in the level of methodological detail provided across individual studies. All studies clearly stated the index modality (radiomics/CT or MRI, whole-slide histopathology, or DNA methylation profiling), the diagnostic target and reference standard (histopathology), and provided basic cohort sizes and model types. However, several TRIPOD-aligned elements [18] were incompletely reported, with variability across studies. For example, patient flow and handling of missing data were incompletely reported or not clearly described in most studies [2,3,21,24,25,26]. Meanwhile, only Yu et al. [22] and Shen et al. [23] provided more structured validation frameworks. Blinding of the index test assessment and reference standard interpretation was rarely reported. Model specification sufficient for reproducibility—such as full hyperparameter disclosure or access to code—was inconsistently described across studies.

External validation was reported only for the CT-based benign–malignant discrimination models by Yu et al. [22] and Shen et al. [23], while all other studies relied exclusively on internal validation or cross-validation approaches. Calibration assessment and decision-analytic clinical utility were reported only by Shen et al. [23], highlighting a substantial gap between discrimination-focused reporting and clinically actionable model evaluation. Explicit algorithmic explainability was limited, with Grad-CAM implementation reported only in Yu et al. [22], while other studies relied on indirect feature-based interpretation or did not report explainability approaches. Data sharing was uncommon, and reproducibility was further limited by the limited availability of publicly accessible datasets or model code across studies; only one study explicitly indicated data availability upon request [25]. A structured TRIPOD/TRIPOD-AI item summary is provided in Table 5.

### 3.9. Reporting Bias Assessment

Publication bias was not assessed using Deeks’ funnel plot asymmetry test because fewer than 10 clinically comparable studies were available for any single diagnostic question with extractable, patient-level test performance data; therefore, the planned analysis was not applicable to the current evidence base.

### 3.10. Certainty of Evidence (GRADE) for Externally Validated CT-Based Benign–Malignant Discrimination

Certainty of evidence for the benign–malignant discrimination task using CT-based AI models with external validation [22,23] (Table 6) was judged to be low overall. Evidence was downgraded for risk of bias (retrospective designs, unclear blinding and participant selection processes, and limited information on flow/timing), and for inconsistency (material differences in discrimination between the two externally validated models, reflecting heterogeneity in model type and feature construction). Imprecision was also considered serious because, although both studies reported confidence intervals for AUC, the evidence base comprises only two studies with different modeling approaches and limited replication across settings. Indirectness was not considered serious because both studies addressed the intended clinical question (preoperative benign vs. malignant parotid tumor discrimination) using histopathologic confirmation. Publication bias could not be formally assessed due to the small number of comparable studies.

## 4. Discussion

Malignant salivary gland tumors represent one of the most diagnostically complex entities in head and neck oncology due to their rarity, histologic diversity, and overlapping morphologic patterns. The integration of AI across radiologic, histopathologic, and molecular domains has been proposed as a strategy to enhance diagnostic precision, reduce interobserver variability, and improve preoperative planning. In this systematic review and functional meta-synthesis, eight studies comprising 1922 patients were analyzed across three technological domains: radiologic AI models [21,22,23,24], digital histopathologic deep-learning systems [3,25,26], and epigenetic methylation-based classifiers [2]. The synthesis demonstrates that while discrimination performance is often high in internal validation, true external validation remains limited, and methodological heterogeneity constrains immediate clinical implementation. This heterogeneity arises from multiple sources, including differences in imaging modalities (CT versus MRI), acquisition protocols, segmentation strategies, and feature extraction pipelines in radiomics studies; variability in staining, scanning resolution, and patch-level versus slide-level analysis in histopathologic models; and differences in molecular platforms, preprocessing workflows, and classification targets in methylation-based approaches. Additionally, substantial variation exists in model architectures (e.g., conventional machine learning versus deep learning), feature selection strategies, and reported outcomes, including binary versus multi-class classification tasks and inconsistent reporting of performance metrics.

The most clinically relevant findings emerged from CT-based externally validated models for benign–malignant discrimination [22,23]. These studies demonstrated moderate-to-high discrimination capacity, suggesting that deep learning applied to contrast-enhanced CT may support preoperative stratification. However, this evidence is currently limited to a small number of externally validated studies. Moreover, calibration metrics, decision-curve analyses, and impact studies were largely absent, highlighting a critical gap between discrimination-focused model development and clinically actionable performance. While many models reported high accuracy or AUC values, key elements required for clinical translation—including calibration, clinical utility assessment, interpretability, and reproducibility—were inconsistently reported or entirely lacking across most studies. Notably, only one of the included studies performed a formal assessment of clinical utility, further limiting the ability to determine the real-world impact of these models on clinical decision-making.

Imaging-based radiomics and deep learning frequently show promising apparent discrimination, yet generalizability across scanners, institutions, and acquisition protocols remains an unresolved challenge [27,28]. This limitation is particularly evident in radiomics models developed in single-center studies with relatively small sample sizes, where restricted population variability and institutional-specific imaging protocols may reduce external validity and hinder clinical translation.

MRI-based radiomics and hybrid models integrating inflammatory biomarkers demonstrated encouraging internal performance [21,24]. The inclusion of systemic inflammatory indices reflects a biologically plausible link between tumor-associated inflammation and malignant potential. Nevertheless, these findings require independent validation, as retrospective dataset partitioning and feature preselection prior to splitting increase susceptibility to overfitting and information leakage [29,30,31,32]. Digital histopathologic convolutional neural networks showed strong internal discrimination across multiple tumor subtypes [3,25,26]. However, performance varied substantially depending on architecture and resolution, underscoring the sensitivity of histopathologic AI to preprocessing decisions and dataset curation. Similar patterns have been observed in broader computational pathology research [29,30]. Importantly, none of the histopathologic models included in this review underwent independent external validation, which substantially limits confidence in their generalizability and clinical applicability.

The most conceptually transformative contribution among the included studies was the application of DNA methylation-based classification for salivary gland tumor taxonomy [2]. Unlike radiologic or morphologic pattern recognition, methylation profiling interrogates epigenetic signatures that reflect tumor lineage, differentiation state, and oncogenic pathway activation. DNA methylation patterns are remarkably stable and tissue-specific, enabling reproducible classification across entities that may be morphologically ambiguous. Balanced accuracy approached near-perfect discrimination under cross-validation conditions; however, this performance should be interpreted cautiously given the absence of independent external validation and the potential for optimistic performance estimates inherent to internal validation frameworks [2].

Epigenetic profiling introduces several advantages. It reduces observer dependence, captures biologic heterogeneity that may not be apparent histologically, and may reveal cryptic entities within morphologically overlapping groups. This aligns with broader oncologic advances in methylation-based tumor classification, particularly in central nervous system neoplasms, where epigenetic classifiers have reshaped diagnostic standards [33,34,35]. In salivary gland pathology, where rare entities are common and interobserver agreement can be moderate, methylation profiling may offer an objective framework for classification refinement. However, further validation is required before clinical integration.

However, molecular classifiers introduce practical considerations, including cost, infrastructure requirements, and turnaround time. External validation in prospective cohorts is necessary to confirm robustness across populations and technical platforms. Molecular classifiers should be viewed as complementary diagnostic layers that refine uncertain cases rather than replace histopathology. Integration of radiologic texture, histologic architecture, and epigenetic signatures within unified multimodal AI frameworks may represent the next evolutionary step in salivary gland tumor diagnostics [31,36,37].

Risk of bias assessment across included studies revealed frequent high risk in analysis domains, largely due to retrospective sampling and incomplete calibration reporting. Reporting transparency was inconsistent, with incomplete adherence to emerging TRIPOD-AI standards [18]. The certainty of evidence for externally validated CT-based discrimination was rated low-to-moderate, whereas certainty for histopathologic and methylation-based models was low, given exclusive internal validation. Quantitatively, only two of the eight included studies provided external validation for a comparable diagnostic task. Meanwhile, the remaining studies relied exclusively on internal validation frameworks, further limiting the strength and generalizability of the evidence base.

This review presents both notable strengths and important limitations. It integrates radiomics, digital pathology, and epigenetic classification within a unified functional interpretative framework. Rather than focusing solely on pooled discrimination metrics, the functional meta-synthesis clarifies how AI operates across hierarchical biological levels: macroscopic radiologic phenotype, microscopic tissue architecture, and molecular epigenetic identity. At the same time, its findings are constrained by the limited number of eligible studies, methodological heterogeneity, and the predominance of internally validated models, which should be considered when interpreting the results.

Moreover, an imbalance across technological domains should be acknowledged. While radiologic and histopathologic AI models were more frequently represented, only a single study addressed biomarker-based (molecular) classification. This disproportion reflects an important gap in the current literature rather than a limitation of the review process, highlighting the relative underdevelopment of biomarker-driven AI approaches in the diagnostics of malignant salivary gland tumors.

An important limitation of this review is the relatively small number of included studies (n = 8), which reflects both the rarity of malignant salivary gland tumors and the early-stage nature of AI applications in this field. This limited evidence base may introduce selection bias, as studies with positive or higher-performing models are more likely to be published and subsequently included. Furthermore, the small number of studies and their heterogeneity restrict the generalizability of the findings across different populations, institutions, and technical settings.

Clinically, AI-based CT models may support surgical planning and preoperative counseling. Histopathologic deep-learning systems may serve as second-opinion tools in diagnostically ambiguous cases. Epigenetic classifiers may have the potential to refine rare or borderline entities and contribute to precision oncology approaches. However, no included system currently meets the criteria for autonomous clinical deployment. This limitation is not only related to validation design, but also to the systematic underreporting of calibration, clinical utility, interpretability, and reproducibility, which remain major unresolved challenges in the field. These methodological limitations have direct practical implications: current AI models should be interpreted as decision-support tools rather than standalone diagnostic systems; they require integration with clinical, radiologic, and histopathologic judgment to ensure safe application in practice. Prospective multicenter validation, standardized reporting, calibration assessment, and clinical impact analyses remain essential.

Future research should prioritize prospective external validation, multimodal integration, and fairness analyses across diverse populations. The convergence of imaging, digital pathology, and molecular epigenetics within interoperable AI ecosystems may ultimately transform diagnostic workflows in salivary gland oncology.

## 5. Conclusions

This systematic review and functional meta-synthesis demonstrates that artificial-intelligence-based models show promising diagnostic performance in malignant salivary gland tumors across radiologic, histopathologic, and epigenetic domains. Externally validated CT-based models achieved moderate-to-high discriminatory capacity for benign–malignant differentiation, suggesting a potential role in preoperative decision-making. However, current evidence remains limited and requires further external validation. Histopathologic deep-learning systems and DNA methylation-based classifiers may contribute to improved diagnostic precision in controlled or internally validated settings, enabling refined subtype classification and molecular taxonomy. However, most models rely on internal validation, and methodological heterogeneity, limited calibration reporting, and high risk of bias reduce the certainty of current evidence. These limitations have important methodological implications. The predominance of retrospective designs and internal validation strategies increases the risk of overfitting, whereby models may capture dataset-specific patterns rather than generalizable diagnostic signals. In addition, the lack of calibration assessment limits the ability to determine whether predicted probabilities accurately reflect true outcome likelihoods, thereby constraining clinical applicability and decision-making reliability. Overall, the current evidence should be considered exploratory and hypothesis-generating rather than confirmatory.

Collectively, these findings indicate that AI should be regarded as an advanced decision-support tool rather than an autonomous diagnostic system. Multicenter prospective validation, strict adherence to TRIPOD-AI reporting standards, and the development of multimodal frameworks integrating imaging, digital pathology, and molecular data are essential before routine clinical implementation. The convergence of radiologic phenotype, histologic architecture, and epigenetic identity represents a promising yet still evolving pathway toward more precise, biologically informed salivary gland tumor diagnostics.

## Figures and Tables

**Table 1:** Study design, population, AI modality, diagnostic task, and validation strategy of included studies.

Author (Year)	Country	Modality	Sample Size (n)	Diagnostic Task	Validation Strategy
Jurmeister et al. (2024) [2]	Germany (multicenter)	DNA methylation profiling + SVM	363	Multi-entity SGT classification	Repeated cross-validation
Schulz et al. (2023) [3]	Germany	Digital histopathology (WSI CNN)	68	Salivary gland carcinoma classification	Train–test split (internal)
He et al. (2022) [21]	China	MRI radiomics + ML	298	Four-class parotid tumor classification	7:3 train–test split
Yu et al. (2023) [22]	China (multicenter)	CT deep learning (CNN)	573	Benign vs. malignant parotid tumors	Training, internal testing, external testing
Shen et al. (2024) [23]	China (multicenter)	CT radiomics (tumor + peritumor)	374	Benign vs. malignant parotid tumors	Training, internal validation, external validation
Committeri et al. (2023) [24]	Italy	MRI radiomics + inflammatory biomarkers	117	WT vs. PA vs. malignant	Train–test split
Sousa-Neto et al. (2025) [25]	Brazil	WSI deep learning (ResNet-50)	83	CXPA vs. PA	Train–test split
Sousa-Neto et al. (2026) [26]	Brazil	WSI deep learning (multiple CNNs)	46	AciCC vs. SC	Train–test split

SGT, salivary gland tumor; SVM, support vector machine; WSI, whole-slide imaging; CNN, convolutional neural network; MRI, magnetic resonance imaging; ML, machine learning; CT, computed tomography; WT, Warthin tumor; PA, pleomorphic adenoma; CXPA, carcinoma ex pleomorphic adenoma; AciCC, acinic cell carcinoma; SC, secretory carcinoma.

**Table 2:** Diagnostic performance metrics of included AI/ML models. External validation results are prioritized when available.

Study	Model/Architecture	Validation Type	AUC (95% CI If Reported)	Accuracy	Sensitivity	Specificity
Jurmeister et al. [2]	SVM methylation classifier	Internal (cross-validation)	Balanced accuracy 0.991 *	NR	NR	NR
Schulz et al. [3]	Inception v3 (CNN)		NR	0.847	Up to 0.85 (recall)	NR
He et al. [21]	XGBoost (Step 1 test)	Internal (test set)	0.826 (NR)	0.899	0.647	0.958
Yu et al. [22]	MobileNet V3 (CNN)	External	0.890 (0.844–0.937)	0.846	0.828	0.860
Shen et al. [23]	Radiomics (Tumor + External2, SVM)	External	0.745 (0.699–0.791)	0.773	0.794	0.714
Committeri et al. [24]	SVM multivariate model	Internal (test set)	NR (ROC reported)	0.86	0.68	0.91
Sousa-Neto et al. [25]	ResNet-50 (CNN)	Internal (test set)	0.97 (NR)	0.93	0.94	0.88
Sousa-Neto et al. [26]	InceptionV3 (best accuracy); VGG16 (highest AUC)	Internal (test set)	0.86 (NR)	0.81	0.90	0.73

* Jurmeister et al. [2] reported mean balanced accuracy for multi-class methylation-based tumor classification. Because the model addressed simultaneous discrimination across multiple histologic entities, conventional binary diagnostic metrics such as AUC, sensitivity, and specificity were not provided in a directly comparable format. NR = Not Reported in the original study. SVM, support vector machine; CNN, convolutional neural network; ROC, Receiver Operating Characteristic

**Table 3:** QUADAS-2 Risk of Bias and Applicability Assessment.

Study	Patient Selection	Index Test	Reference Standard	Flow and Timing	Applicability: Patients	Applicability: Index Test	Applicability: Reference Standard
Jurmeister et al. [2]	High	High	Low	Unclear	Unclear	Low	Low
Schulz et al. [3]	High	High	Low	Unclear	Unclear	Low	Low
He et al. [21]	High	High	Low	Unclear	Unclear	Low	Low
Yu et al. [22]	High	High	Low	Unclear	Low	Low	Low
Shen et al. [23]	High	High	Low	Unclear	Low	Low	Low
Committeri et al. [24]	High	High	Low	Unclear	Unclear	Low	Low
Sousa-Neto et al. [25]	High	High	Low	Unclear	Unclear	Low	Low
Sousa-Neto et al. [26]	High	High	Low	Unclear	Unclear	Low	Low

**Table 4:** PROBAST Domain-Level Risk of Bias Assessment.

Study	Participants	Predictors	Outcome	Analysis
Jurmeister et al. [2]	High	Low	Low	High
Schulz et al. [3]	High	Low	Low	High
He et al. [21]	High	Low	Low	High
Yu et al. [22]	High	Low	Low	High
Shen et al. [23]	High	Low	Low	High
Committeri et al. [24]	High	Low	Low	High
Sousa-Neto et al. [25]	High	Low	Low	High
Sousa-Neto et al. [26]	High	Low	Low	High

**Table 5:** TRIPOD/TRIPOD-AI reporting elements across included studies.

TRIPOD Element	Jurmeister et al. [2]	Schulz et al. [3]	He et al. [21]	Yu et al. [22]	Shen et al. [23]	Committeri et al. [24]	Sousa-Neto et al. [25]	Sousa-Neto et al. [26]
Population eligibility/setting described	Partial	Partial	Partial	Yes	Yes	Partial	Partial	Partial
Reference standard (histopathology) stated	Yes	Yes	Yes	Yes	Yes	Yes	Yes	Yes
Sample size reported	Yes	Yes	Yes	Yes	Yes	Yes	Yes	Yes
Index test/model description (modality + algorithm)	Yes	Yes	Yes	Yes	Yes	Yes	Yes	Yes
Model development details sufficient for replication	Partial	No/limited	Partial	Partial	Partial	Partial	Partial	Partial
Handling of missing data reported	Not reported	Not reported	Not reported	Not reported	Not reported	Not reported	Not reported	Not reported
Validation approach reported	Yes	Yes	Yes	Yes	Yes	Yes	Yes	Yes
External validation performed	No	No	No	Yes	Yes	No	No	No
Discrimination metrics reported (e.g., AUC/accuracy)	Yes	Yes	Yes	Yes	Yes	Yes	Yes	Yes
PPV/NPV reported for the main model	No	No	No/unclear	Yes	Yes	Unclear/limited	Partial (NPV/precision)	No
Calibration metrics reported (e.g., calibration curve/Brier)	No	No	No	No	Yes	No	No	No
Clinical utility analysis reported (e.g., DCA/net benefit)	No	No	No	No (NRI/IDI reported)	Yes	No	No	No
Explicit interpretability method reported (e.g., Grad-CAM/SHAP)	No	No	No	Yes (Grad-CAM)	No	No	No	No (stated not implemented)
Data/code availability statement	No/unclear	No/unclear	No/unclear	No/unclear	No/unclear	No/unclear	Yes (upon request)	No/unclear

AUC, area under the curve; DCA, decision curve analysis; NPV, negative predictive value; PPV, positive predictive value; TRIPOD, Transparent Reporting of a multivariable prediction model for Individual Prognosis or Diagnosis; TRIPOD-AI, AI extension (under development guidance).

**Table 6:** GRADE summary (narrative) for externally validated CT-based AI models (benign vs. malignant parotid tumors).

Outcome/Evidence Base	Risk of Bias	Inconsistency	Indirectness	Imprecision	Overall Certainty
Benign vs. malignant parotid tumors (CT-based AI models with external validation): Yu et al. [22] external AUC 0.890 (95% CI 0.844–0.937), accuracy 0.846, sensitivity 0.828, specificity 0.860, PPV 0.716, NPV 0.917; Shen et al. [23] external AUC 0.745 (95% CI 0.699–0.791) for Tumor + External2 radiomics (SVM), accuracy 0.773, sensitivity 0.794, specificity 0.714, PPV 0.885, NPV 0.555; calibration curves/Brier scores and DCA reported by Shen et al. [23].	Serious (downgrade 1)	Serious (downgrade 1)	Not serious	Serious (downgrade 1)	Low

## Data Availability

The original contributions presented in this study are included in the article/Supplementary Materials. Further inquiries can be directed to the corresponding author.

## Supplementary Materials

The following supporting information can be downloaded at https://www.mdpi.com/article/10.3390/medsci14020183/s1. Table S1: Complete Search Strategies for Each Database.
