# Supplementary material for: Artificial-Intelligence-Based Radiologic, Histopathologic, and Molecular Models for the Diagnosis and Classification of Malignant Salivary Gland Tumors: A Systematic Review and Functional Meta-Synthesis
Source: Med Sci (Basel). 2026 Apr 5;14(2):183. doi: 10.3390/medsci14020183 (PMC13108205; doi:10.3390/medsci14020183)
Supplement: Supplementary file 1 [file medsci-14-00183-s001.zip › medsci-4207856-supplementary.pdf]

medical sciences

Supplementary Materials: Artificial-Intelligence-Based Radiologic, Histopathologic, and Molecular Models for the Diagnosis and Classification of Malignant Salivary Gland Tumors: A Systematic Review and Functional Meta-Synthesis

Carlos M. Ardila [1,2,*], Eliana Pineda-Vélez [2,3], Anny M. Vivares-Builes [2,3] and Alejandro I. Díaz-Laclaustra [4]

Table S1. Search strategies for the systematic review.

Database	Search strategy
PubMed (MEDLINE via PubMed)	("Salivary Gland Neoplasms"[Mesh] OR "Parotid Neoplasms"[Mesh] OR salivary gland neoplasm*[tiab] OR salivary gland tumor*[tiab] OR salivary gland cancer*[tiab] OR parotid neoplasm*[tiab] OR parotid tumor*[tiab] OR salivary carcinoma*[tiab] OR "mucoepidermoid carcinoma"[tiab] OR "adenoid cystic carcinoma"[tiab] OR "salivary duct carcinoma"[tiab] OR "carcinoma ex pleomorphic adenoma"[tiab]) AND ("Artificial Intelligence"[Mesh] OR "Machine Learning"[Mesh] OR "Deep Learning"[Mesh] OR artificial intelligence[tiab] OR machine learning[tiab] OR deep learning[tiab] OR neural network*[tiab] OR convolutional neural network*[tiab] OR CNN[tiab] OR radiomic*[tiab] OR radiomics[tiab] OR "digital pathology"[tiab] OR "whole slide imaging"[tiab] OR WSI[tiab] OR (methylation[tiab] AND (classifier*[tiab] OR "machine learning"[tiab] OR "artificial intelligence"[tiab])))
Embase (Ovid)	(<br>('salivary gland tumor'/exp OR 'salivary gland neoplasm'/exp OR 'parotid tumor'/exp OR 'parotid neoplasm'/exp)<br>OR<br>('salivary gland tumor':ti,ab,kw OR<br>'salivary gland neoplasm':ti,ab,kw OR<br>'salivary gland cancer':ti,ab,kw OR<br>'parotid tumor':ti,ab,kw OR<br>'parotid neoplasm':ti,ab,kw OR<br>'salivary carcinoma':ti,ab,kw OR<br>'mucoepidermoid carcinoma':ti,ab,kw OR<br>'adenoid cystic carcinoma':ti,ab,kw OR<br>'salivary duct carcinoma':ti,ab,kw OR<br>'carcinoma ex pleomorphic adenoma':ti,ab,kw)<br>)<br>AND<br>(<br>('artificial intelligence'/exp OR 'machine learning'/exp OR 'deep learning'/exp)<br>OR<br>('artificial intelligence':ti,ab,kw OR<br>'machine learning':ti,ab,kw OR<br>'deep learning':ti,ab,kw OR<br>'neural network':ti,ab,kw OR<br>'convolutional neural network':ti,ab,kw OR<br>CNN:ti,ab,kw OR<br>radiomic*:ti,ab,kw OR

	radiomics:ti,ab,kw OR<br>'digital pathology':ti,ab,kw OR<br>'whole slide imaging':ti,ab,kw OR<br>WSI:ti,ab,kw OR<br>(methylation:ti,ab,kw AND (classifier:ti,ab,kw OR 'machine learning':ti,ab,kw OR 'artificial intelligence':ti,ab,kw))<br>)<br>)
Scopus	(TITLE-ABS-KEY(salivary AND gland AND (tumor OR neoplasm OR carcinoma))<br>OR TITLE-ABS-KEY(parotid AND (tumor OR neoplasm OR carcinoma))<br>OR TITLE-ABS-KEY("mucoepidermoid carcinoma")<br>OR TITLE-ABS-KEY("adenoid cystic carcinoma")<br>OR TITLE-ABS-KEY("salivary duct carcinoma")<br>OR TITLE-ABS-KEY("carcinoma ex pleomorphic adenoma"))<br>AND<br>(TITLE-ABS-KEY("artificial intelligence")<br>OR TITLE-ABS-KEY("machine learning")<br>OR TITLE-ABS-KEY("deep learning")<br>OR TITLE-ABS-KEY("neural network")<br>OR TITLE-ABS-KEY("convolutional neural network")<br>OR TITLE-ABS-KEY(CNN)<br>OR TITLE-ABS-KEY(radiomics)<br>OR TITLE-ABS-KEY(radiomic)<br>OR TITLE-ABS-KEY("digital pathology")<br>OR TITLE-ABS-KEY("whole slide imaging")<br>OR TITLE-ABS-KEY(WSI)<br>OR TITLE-ABS-KEY(methylation))
